# Supplementary material for: Genome-wide association study of cerebellar volume provides insights into heritable mechanisms underlying brain development and mental health
Source: Commun Biol. 2022 Jul 16;5:710. doi: 10.1038/s42003-022-03672-7 (PMC9288439; doi:10.1038/s42003-022-03672-7)
Supplement: Supplementary file 2 — Supplementary Information [file 42003_2022_3672_MOESM2_ESM.pdf]

Supplementary Information

Genome-wide association study of cerebellar volume provides insights into heritable mechanisms underlying brain development and mental health

Elleke Tissink, Siemon C. de Lange, Jeanne E. Savage, Douglas P. Wightman, Christiaan A. de Leeuw, Kristen M. Kelly, Mats Nagel, Martijn P. van den Heuvel, Danielle Posthuma

Supplementary Notes

1. *Comparison results with Smith et al and Zhao et al…………………………………………………..1*
2. *Comparison results with Chambers et al……………………………………………………………..2*
3. *Genetic correlations with cerebral and subcortical volume.………………………………………2*

Supplementary Figures

1. *Supplementary Figure 1……………………………………………………………………………..3*
2. *Supplementary Figure 2……………………………………………………………………………..4*
3. *Supplementary Figure 3……………………………………………………………………………..5*
4. *Supplementary Figure 4……………………………………………………………………………..6*

Supplementary References

Supplementary Notes

1. Comparison results with Smith et al and Zhao et al

Smith *et al*[1] and Zhao *et al*[2] have provided *en masse* summary statistics, including for the volume of cerebellar vermal lobules and lateral cerebellar grey and white matter (total cerebellar volume was not investigated). The reported h^2_{SNP} estimated with GCTA-GREML varied largely across cerebellum subregions in the Zhao *et al* publication (M = 64.55%, SD = 10.64%), whereas lower and more stable LDSC-based h^2_{SNP} estimates were observed (M = 27.95%, SD = 0.35%) by Smith *et al*. Smith *et al* do not provide any post-GWAS investigation, but Zhao *et al* report 19 non-overlapping loci, 69 mapped genes and none gene-sets to be significantly associated with at least one cerebellar volume subregion. We also observe 10 of those loci and 35 of those genes for total cerebellar volume, but additionally report 20 and 191 novel loci and genes respectively. This increase is likely due to our significantly larger sample size (N = 27,486) compared to Zhao *et al* (N = 19,629) and our cerebellum-specific gene-mapping protocol instead of using all brain-based eQTL and chromatin interaction reference data available with default settings by Zhao *et al*.

2. Comparison results with Chambers et al

Chambers *et al*[3] provides a GWAS of total cerebellar grey matter volume and reports 29 loci from GCTA-COJO (Table 1 of ref 10). Since we included a different phenotype (total cerebellar grey and white matter volume) and defined loci in FUMA, comparing loci one-on-one can be arbitrary. However, 18 of the extended LD-ranges described by Chambers *et al* overlap with our loci and from our 29 loci, 14 are also reported by Chambers *et al* (Supplementary Table 19). Our LDSC-based h^2_{SNP} estimate (39.8%, SE = 3.14%) is comparable with Chambers *et al* GCTA-GREML-based estimate (45.3-46.8%). The authors of ref 10 state that 732 gene transcripts overlap with the complete extended-LD regions of the 33 index SNPs and map 14 genes via cerebellum-specific eQTLs, whereas we map 189 genes based on <1MB genomic proximity and 32 genes via cerebellum-specific eQTLs. We further provide novel results by stratifying the heritability, defining and comparing the genetic architecture using MiXeR, and finemaping all identified loci.

The largest difference between our study and ref 10 lies within the gene-based follow up that our study provides. We perform a gene-based GWAS and identify 85 significant genes associated with cerebellar volume. We use the gene-based summary statistics for four novel gene-set analyses: cerebellar cell-type specificity analysis, temporal cerebellar gene-expression analysis, biological pathway analysis and an additional evolutionary gene-set analysis. By doing so, we show nominal significant associations between cerebellar volume gene associations on the one hand, and astrocyte specific gene-expression and prenatal cerebellar gene-expression on the other hand.

Another difference between ref 10 and our study is related to genetic correlation analyses. Chambers *et al* do not to find nominal significant global genetic correlation between total cerebellar grey matter volume and psychiatric disorders (schizophrenia, bipolar, major depression, ASD and ADHD), but report an undirected pleiotropic relationship in 8 GWAS signals with a psychiatric phenotype. We added neurodegenerative disorders given the phenotypic relevance in previous literature. We report a nominal significant global genetic correlation between total cerebellar grey and white matter volume and ADHD, and directed local genetic correlations between total cerebellar grey and white matter volume and schizophrenia, Alzheimer’s and Parkinson’s Disease. We provide additional colocalization results to even zoom in the level of a shared causal variant between these traits.

3. Genetic correlations cerebellar volume with cerebral and subcortical volume

We followed up on comparing the genetic architecture of cerebellar volume with cerebral and subcortical volume by estimating global (Supplementary Data 20) and local (Supplementary Data 21) genetic correlations (r_g). Cerebellar and cerebral volume were significantly genetically correlated (r_g = -0.47, SE = 0.04, $p = 1.35\times10^{-26}$). Note that, by correcting the cerebral and cerebellar volume GWAS for total brain volume, we correlated SNP effects between relative volumetric phenotypes. The negative r_g sign is expected from this correction and opposing effect directions can’t be interpreted in an absolute volumetric manner. On a locus level, we observed a significantly correlated locus after Bonferroni

correction ($p < 7.38\times10^{-6}$) between cerebellar and cerebral volume on chromosome 12 (101,857,796-102,961,329, corr = -1.07, $p = 3.13\times10^{-8}$). Interestingly, a neighboring locus on chromosome 12 (BP 99,816,842-101,857,521) showed significant r_g between subcortical and cerebellar volume (corr = -1.03, $p = 2.00\times10^{-9}$). These correlated loci overlap with a genome-wide significant locus identified in this study and may contain variants with distributed effects on the volume of the three major brain structures, whereas the other identified loci seem more cerebellar volume-specific. Moreover, we did not find global r_g significantly different from zero between subcortical volume and either cerebellar (r_g = 0.03, SE = 0.05, $p = 0.59$) or cerebral volume (r_g = -0.08, SE = 0.05, $p = 0.09$), suggesting a more distinct genetic architecture of subcortical volume compared to cerebral and cerebellar volume.

Supplementary Figures

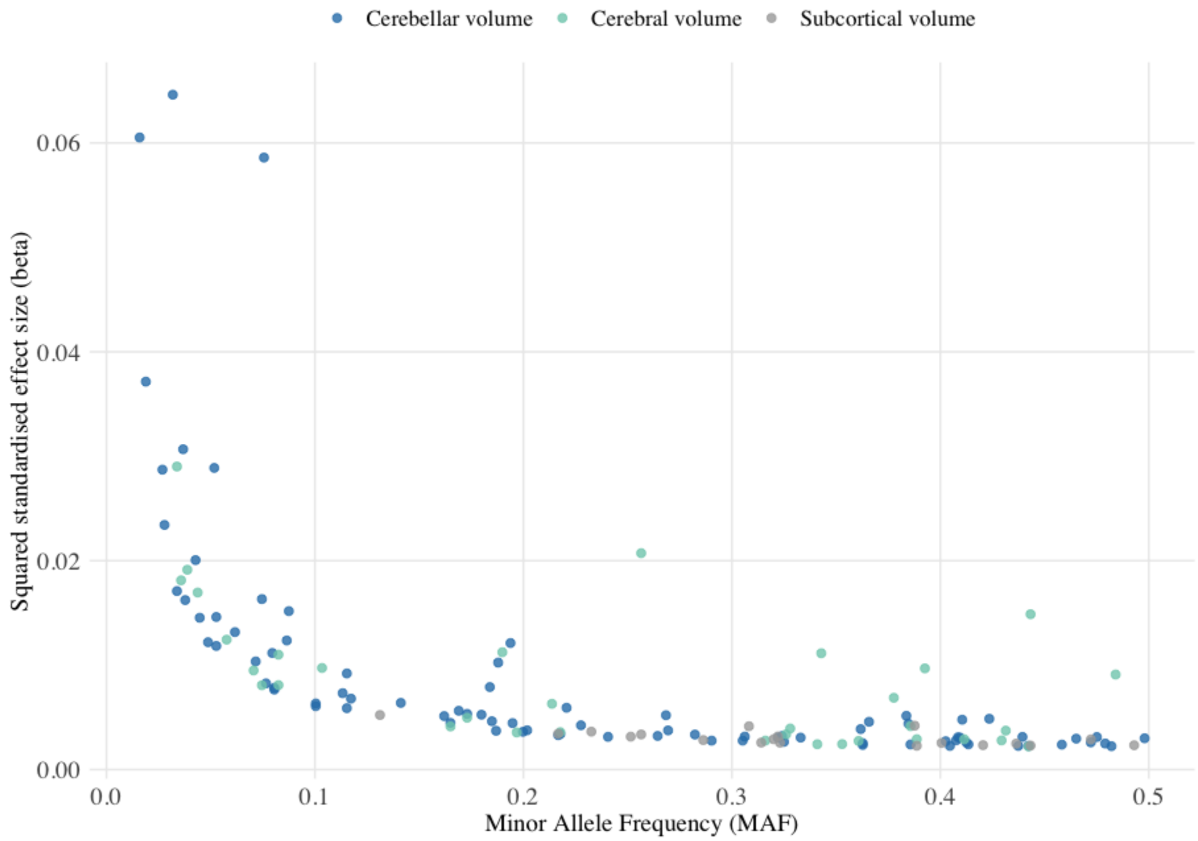

Supplementary Figure 1. Scatter plot of MAF and squared standardized effect sizes (Beta) for cerebellar, cerebral, and subcortical volume independent significant SNPs.

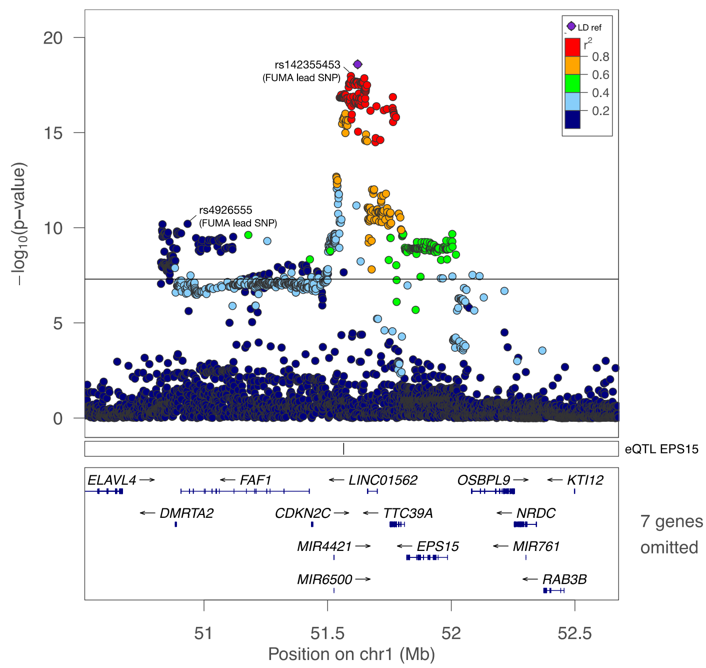

a)

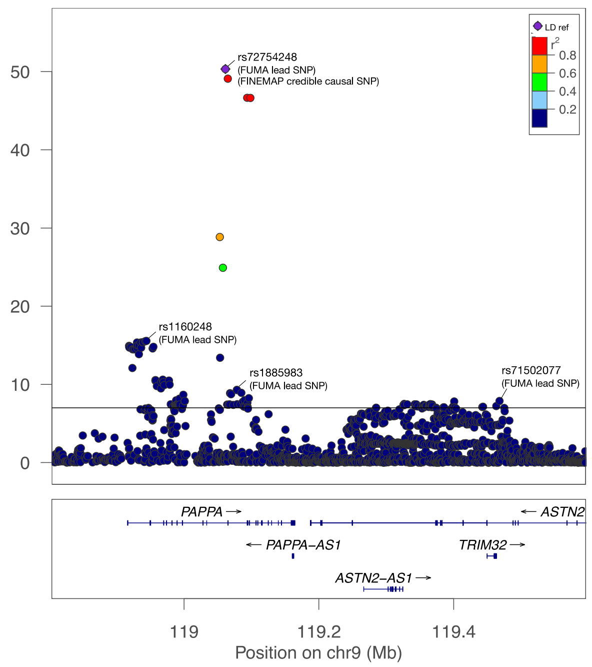

b)

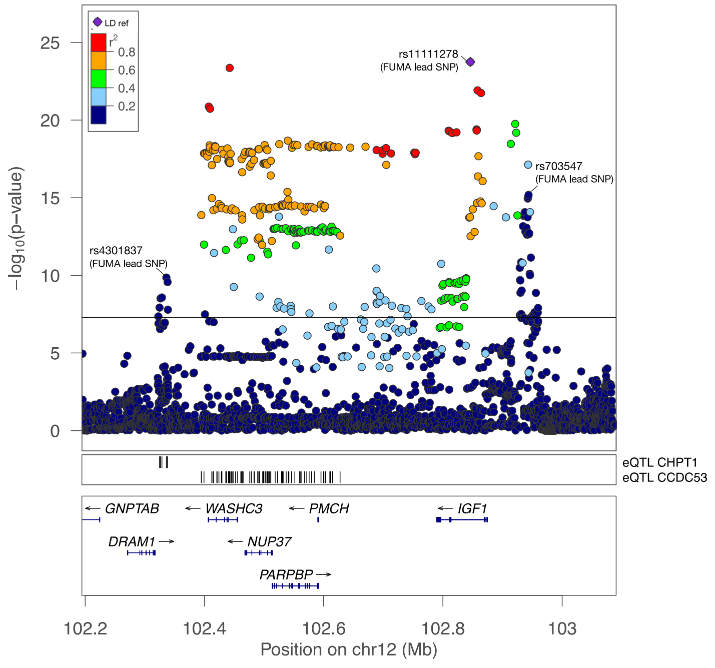

c)

Supplementary Figure 2. LocusZoom plots for the three most significant loci in our cerebellar-volume GWAS on chromosome 1 (a), 9 (b) and 12 (c). FUMA lead SNPs, FINEMAP credible causal SNPs (PIP > 0.95) and/or eQTLs for cerebellar tissue are indicated if applicable.

a)

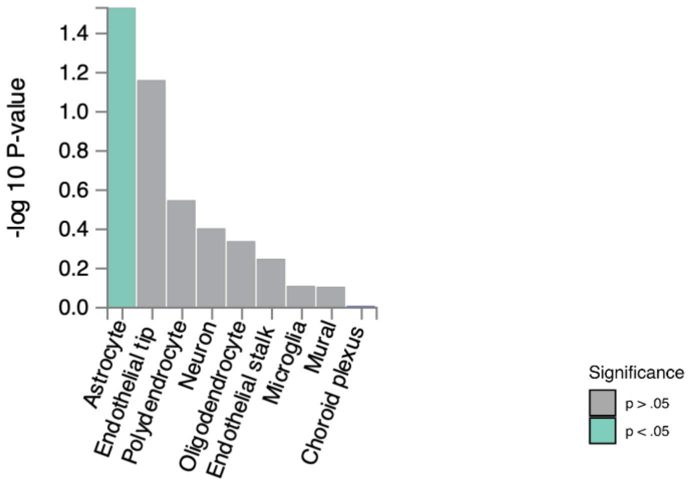

b)

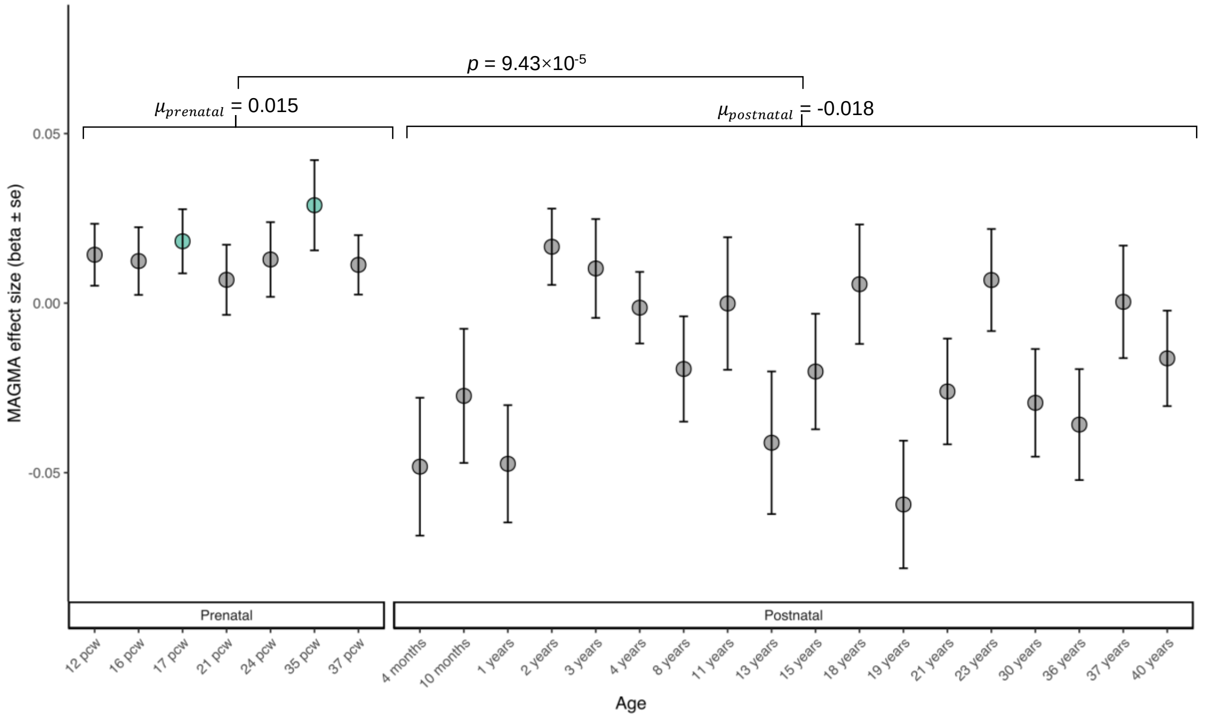

Supplementary Figure 3. Results from two gene-set analysis approaches: a) Gene-expression in cell types from cerebellar mouse tissue (DropViz database in FUMA) was nominally associated with the cerebellar volume gene-based GWAS sumstats in astrocytes, but this did not survive Bonferroni correction (Supplementary Data 12). b) Cerebellar gene-expression in donors from different developmental stages (Brainspan database) was nominally associated with the cerebellar volume gene-based GWAS sumstats at 17 and 35 postconceptual weeks, but these did not survive Bonferroni correction. We compared the average effect (Beta's and standard errors are plotted; see Supplementary Data 10) of gene-expression between pre- and postnatal timepoints, and observed a highly significant difference.

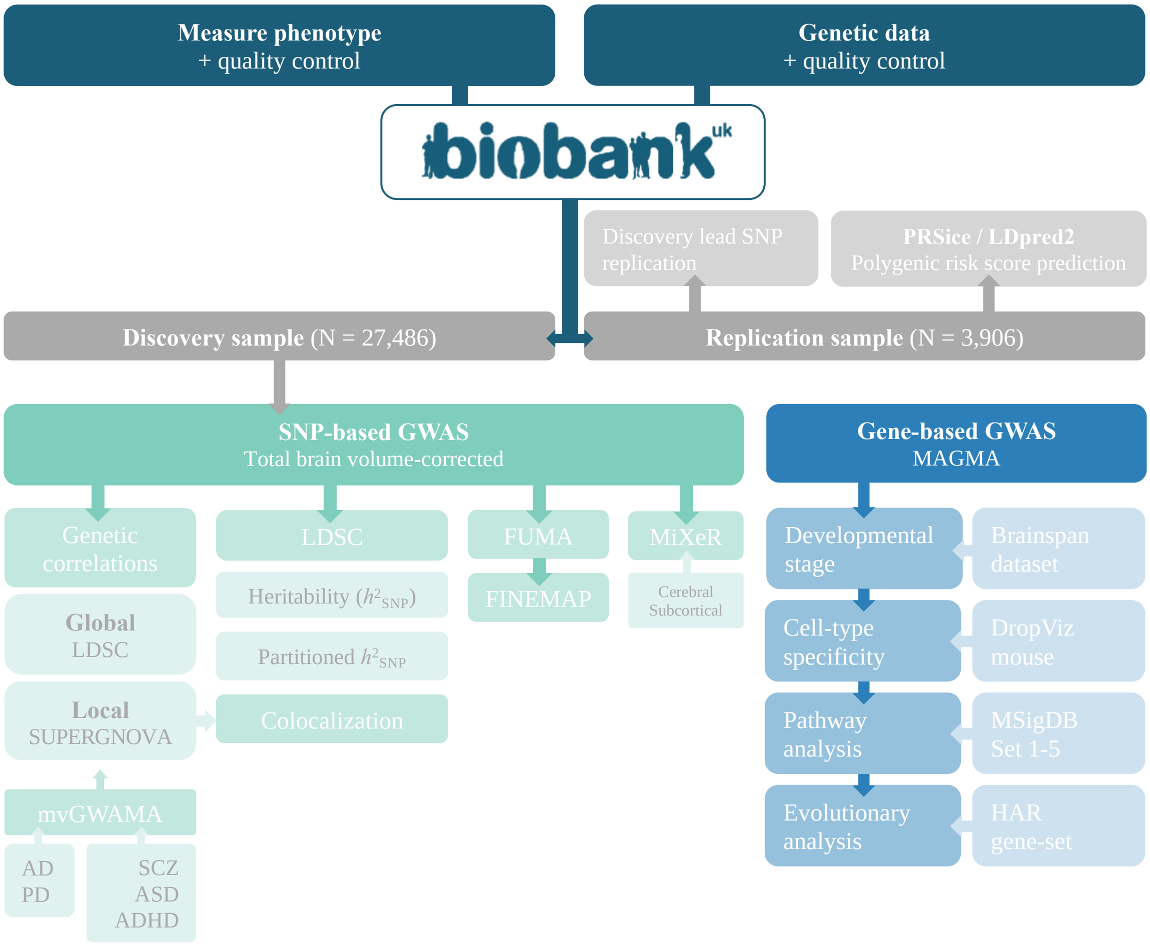

Supplementary Figure 4. Flowchart of all data and methods used to obtain results as presented in this study. For more detailed information see Methods.

Supplementary References

1. Smith, S. M. *et al.* An expanded set of genome-wide association studies of brain imaging phenotypes in UK Biobank. *Nat. Neurosci.* **24**, 737–745 (2021).
2. Zhao, B. *et al.* Genome-wide association analysis of 19,629 individuals identifies variants influencing regional brain volumes and refines their genetic co-architecture with cognitive and mental health traits. *Nat. Genet.* **51**, 1637–1644 (2019).
3. Chambers, T. *et al.* Genetic common variants associated with cerebellar volume and their overlap with mental disorders: a study on 33,265 individuals from the UK-Biobank. *Mol. Psychiatry* (2022). doi:10.1038/s41380-022-01443-8
