# Supplementary material for: Genome-wide association study of cerebellar volume provides insights into heritable mechanisms underlying brain development and mental health
Source: Commun Biol. 2022 Jul 16;5:710. doi: 10.1038/s42003-022-03672-7 (PMC9288439; doi:10.1038/s42003-022-03672-7)
Supplement: Supplementary file 3 — Description of Additional Supplementary Files [file 42003_2022_3672_MOESM3_ESM.pdf]

# Description of Additional Supplementary Files

**File Name:** Supplementary Data

**Description:**

File name: Supplementary Data 1
Description: Partitioned heritability of cerebellar volume

File name: Supplementary Data 2
Description: MiXeR results of cerebellar, cerebral and subcortical volume

File name: Supplementary Data 3
Description: Enrichment of cerebellar volume candidate SNPs in chromatin states, RDB scores, genomic loation

File name: Supplementary Data 4
Description: Overview of cerebellar volume exonic nonsynonymous SNPs

File name: Supplementary Data 5
Description: FUMA output for cerebellar volume candidate SNPs

File name: Supplementary Data 6
Description: Cerebellar volume loci and lead SNPs as identified by FUMA

File name: Supplementary Data 7
Description: FINEMAP results for cerebellar volume loci

File name: Supplementary Data 8
Description: FUMA positional and eQTL gene mapping for cerebellar volume

File name: Supplementary Data 9
Description: MAGMA results for cerebellar volume genome-wide significant genes

File name: Supplementary Data 10
Description: MAGMA gene-property analysis results for BrainSpan gene-expression

File name: Supplementary Data 11
Description: MAGMA gene-set analysis results for biological pathways

File name: Supplementary Data 12

Description: FUMA cell-type specificity results

File name: Supplementary Data 13
Description: Global genetic correlations between cerebellar volume and disorders

File name: Supplementary Data 14
Description: Local genetic correlations between cerebellar volume and disorders

File name: Supplementary Data 15
Description: Colocalization results for correlated loci

File name: Supplementary Data 16
Description: PRSice and LDpred2 results for polygenic score performance

File name: Supplementary Data 17
Description: Association of discovery lead SNPs in replication sample

File name: Supplementary Data 18
Description: Sample quality control overview

File name: Supplementary Data 19
Description: Loci discovery comparison with Chambers et al

File name: Supplementary Data 20
Description: Global genetic correlations between cerebellar, cerebral and subcortical volume

File name: Supplementary Data 21
Description: Local genetic correlations between cerebellar, cerebral and subcortical volume
