# Supplementary material for: Genome-wide association study of cerebellar volume provides insights into heritable mechanisms underlying brain development and mental health
Source: Commun Biol. 2022 Jul 16;5:710. doi: 10.1038/s42003-022-03672-7 (PMC9288439; doi:10.1038/s42003-022-03672-7)
Supplement: Supplementary file 5 — Reporting Summary [file 42003_2022_3672_MOESM5_ESM.pdf]

nature research

Corresponding author(s): Danielle Posthuma

Last updated by author(s): Jun 13, 2022

Reporting Summary

Nature Research wishes to improve the reproducibility of the work that we publish. This form provides structure for consistency and transparency in reporting. For further information on Nature Research policies, see our Editorial Policies and the Editorial Policy Checklist.

Statistics

For all statistical analyses, confirm that the following items are present in the figure legend, table legend, main text, or Methods section.

n/a	Confirmed	
☐	☒	The exact sample size (*n*) for each experimental group/condition, given as a discrete number and unit of measurement
☒	☐	A statement on whether measurements were taken from distinct samples or whether the same sample was measured repeatedly
☐	☒	The statistical test(s) used AND whether they are one- or two-sided<br>*Only common tests should be described solely by name; describe more complex techniques in the Methods section.*
☐	☒	A description of all covariates tested
☐	☒	A description of any assumptions or corrections, such as tests of normality and adjustment for multiple comparisons
☐	☒	A full description of the statistical parameters including central tendency (e.g. means) or other basic estimates (e.g. regression coefficient) AND variation (e.g. standard deviation) or associated estimates of uncertainty (e.g. confidence intervals)
☐	☒	For null hypothesis testing, the test statistic (e.g. *F*, *t*, *r*) with confidence intervals, effect sizes, degrees of freedom and *P* value noted<br>*Give P values as exact values whenever suitable.*
☒	☐	For Bayesian analysis, information on the choice of priors and Markov chain Monte Carlo settings
☒	☐	For hierarchical and complex designs, identification of the appropriate level for tests and full reporting of outcomes
☐	☒	Estimates of effect sizes (e.g. Cohen's *d*, Pearson's *r*), indicating how they were calculated

Our web collection on statistics for biologists contains articles on many of the points above.

Software and code

Policy information about availability of computer code

Data collection	No software was used for data collection purposes.
Data analysis	The following standard software packages were used for the analyses described in the current manuscript:<br>MAGMA: In-house developed software (de Leeuw et al., 2015) was used to conduct gene and gene-set analysis.<br>FUMA: In-house developed online platform for functional annotation of GWAS results (Watanabe et al., 2017).<br>mvGWAMA: In-house developed software (https://github.com/Kyoko-wtnb/mvGWAMA) was used to meta-analyse GWAS summary statistics.<br>LDSC: software (Bulik-Sullival et al., 2015) was used to estimate heritability and global genetic correlations.<br>SUPERGNOVA: software (Zhang et al., 2021) was used to estimate local genetic correlations between traits.<br>COLOC: software (Giambortolomei et al., 2014) was used to estimate the probability of a shared causal variant between traits.<br>FINEMAP: software (Benner et al., 2016) to identify most probable causal variants.<br>MiXeR: software (Holland et al., 2020) to estimate genetic architecture (polygenicity and discoverability).<br>PRSice-2: software (Choi et al., 2019) to compute polygenic scores in replication sample.<br>LDpred2: software (Privé et al., 2020) to compute polygenic scores in replication sample.<br>PLINK 1.9: software (Chang et al., 2015) used for performing genome-wide association analysis.<br>FLASHPCA: software (Abraham et al., 2017) to perform genetic principal component analysis.

For manuscripts utilizing custom algorithms or software that are central to the research but not yet described in published literature, software must be made available to editors and reviewers. We strongly encourage code deposition in a community repository (e.g. GitHub). See the Nature Research guidelines for submitting code & software for further information.

Data

Policy information about availability of data

All manuscripts must include a data availability statement. This statement should provide the following information, where applicable:

- Accession codes, unique identifiers, or web links for publicly available datasets
- A list of figures that have associated raw data
- A description of any restrictions on data availability

In the present study we made use of the publicly available gene expression data and scRNA-seq data as currently implemented in FUMA (GTex, BRAINEAC, DropViz), gene-sets (MSigDB, BrainSpan), brain genes (SynGO), HAR genes (Doan et al., 2016), as well as GWAS summary statistics. URLs are provided in the manuscript.

Field-specific reporting

Please select the one below that is the best fit for your research. If you are not sure, read the appropriate sections before making your selection.

☒ Life sciences ☐ Behavioural & social sciences ☐ Ecological, evolutionary & environmental sciences

For a reference copy of the document with all sections, see nature.com/documents/nr-reporting-summary-flat.pdf

Life sciences study design

All studies must disclose on these points even when the disclosure is negative.

Sample size	We made use of data collected by an external source (UK biobank). The sample size consists of all individuals that remain after quality control of the data and exclusion of withdrawn subjects. Detailed information on the samples used, as well as the exclusion/inclusion criteria, are provided in the Methods, Section: Sample.
Data exclusions	See Methods. We excluded UKB participants from further analyses if they had missing phenotypic data, did not pass standard quality control or withdrew their consent to participate in the UK biobank study (section: Sample).
Replication	We applied an internal validation approach, using a hold-out sample. We explicitly examined the robustness of discovery effects using polygenic scoring and discovery lead SNP replication rate, see Methods.
Randomization	NA
Blinding	NA

Reporting for specific materials, systems and methods

We require information from authors about some types of materials, experimental systems and methods used in many studies. Here, indicate whether each material, system or method listed is relevant to your study. If you are not sure if a list item applies to your research, read the appropriate section before selecting a response.

Materials & experimental systems

n/a	Involved in the study	
☒	☐	Antibodies
☒	☐	Eukaryotic cell lines
☒	☐	Palaeontology and archaeology
☒	☐	Animals and other organisms
☐	☒	Human research participants
☒	☐	Clinical data
☒	☐	Dual use research of concern

Methods

n/a	Involved in the study	
☒	☐	ChIP-seq
☒	☐	Flow cytometry
☐	☒	MRI-based neuroimaging

Human research participants

Policy information about studies involving human research participants

Population characteristics	The discovery sample (N = 27,486) was aged M = 63.55 (SD = 7.52) years with 52.50% females and the replication sample (N = 3,906) was aged M = 64.91 (SD = 7.30) years with 54.58% females.
Recruitment	We utilized data collected previously by UK biobank. All individuals included in the study provided written informed consent, and the study was approved by the concerned ethical committee.
Ethics oversight	NHS Health Research Authority provided ethics approval for the UKB study

Note that full information on the approval of the study protocol must also be provided in the manuscript.

Magnetic resonance imaging

Experimental design

Design type	No dynamic MRI data collection was performed, only cross-sectional structural brain imaging
Design specifications	NA
Behavioral performance measures	NA

Acquisition

Imaging type(s)	Structural brain imaging
Field strength	3 Tesla
Sequence & imaging parameters	T1: 4:54 minute duration, 1x1x1 mm voxel size, 208x256x256 matrix, 3D MPRAGE, sagittal, in-plane acceleration factor (R)=2, T1/TR=800/2000 ms<br>T2 FLAIR: 5:52 minute duration, 1.05x1.0x1.0mm voxel size, 192x256x256 matrix, FLAIR, 3D SPACE, sagittal, in-plane acceleration factor (R)=2, partial Fourier (PR) 7/8, fat sat, T1/TR = 1800/5000 ms, elliptical
Area of acquisition	Whole brain scan

Diffusion MRI ☐ Used ☒ Not used

Preprocessing

Preprocessing software	The UKB Imaging Team developed a fully automated processing pipeline primarily based around FSL software (Jenkinson et al., 2012). For more details, see UK Biobank Brain Imaging Documentation (Smith et al., 2020).
Normalization	non-linear warping to the MNI152 brain template (FSL FNIRT)
Normalization template	standard MNI152 brain template
Noise and artifact removal	gradient distortion correction
Volume censoring	defacing, brain extraction (FSL BET), segmentation and extraction of brain structure volumes

Statistical modeling & inference

Model type and settings	Extracted total grey and white matter cerebellar volume from structural MRI data was used in linear regression models in GWAS
Effect(s) tested	*Define precise effect in terms of the task or stimulus conditions instead of psychological concepts and indicate whether ANOVA or factorial designs were used.*

Specify type of analysis: ☐ Whole brain ☒ ROI-based ☐ Both

Anatomical location(s)	automatic subcortical segmentation as performed by UK Biobank Imaging Team: grey + white cerebellar volume, left + right hemispheres
Statistic type for inference<br>(See Eklund et al. 2016)	Standard linear regression model for testing associations between SNPs and total grey and white matter cerebellar volume phenotypes derived from the T1-weighted scan
Correction	Bonferroni correction for the number of independent variants that were tested in association with total cerebellar volume (P<5x10-8)

Models & analysis

n/a	Involved in the study
☒	☐ Functional and/or effective connectivity
☒	☐ Graph analysis
☒	☐ Multivariate modeling or predictive analysis
